# Unmasking the haemostatic potential of quotidian plastics: upon first glance

**DOI:** 10.3389/fphar.2026.1870771

**Published:** 2026-07-29

**Authors:** Rocío Gomez, Yesenia I. Martinez-Jimenez, Marco Antonio Meraz Ríos

**Affiliations:** 1 Departamento de Toxicología, Centro de Investigación y de Estudios Avanzados del Instituto Politécnico Nacional, Mexico City, Mexico; 2 Departamento de Biomedicina Molecular, Centro de Investigación y de Estudios Avanzados del Instituto Politécnico Nacional, Mexico City, Mexico

**Keywords:** acrylamide, bisphenols, cardiotoxicity, coagulation, microplastics, nanoplastics, phthalates

## Abstract

Acrylamide, bisphenols, phthalates, and micro- and nanoplastics are pervasive environmental pollutants with well-documented carcinogenic, neurotoxic, and reproductive effects. Nonetheless, their potential contribution to cardiovascular diseases remains strikingly unrecognised, despite the rising incidence of their clinical manifestations in younger populations and growing evidence of their effects on haemostasis and thrombosis. Single- and dual-antiplatelet therapy only partially controls the prothrombotic phenotype driven by thromboinflammation, whilst the role of plastics as platelet aggregation enhancers remains an under-addressed gap in current cardiovascular disease prevention strategies. This mini-review synthesises 11 years of *ex vivo* human and *in vivo* rodent evidence on the haemostatic effects of plastics, with attention to platelet aggregation, blood coagulation parameters, and their possible mechanisms. Bisphenols dominate the available literature, skewing the evidence towards their haemostatic effects relative to findings on acrylamide and phthalates. Regarding micro- and nanoplastics, most evidence has come from microplastics, owing to analytical challenges in quantifying nanoplastics. Because exposure to plastics and their complex chemical mixtures remains largely unregulated, the collated evidence on their cardiovascular effects should prompt regulatory action. Our review also highlights the research gaps to be prioritised in future toxicological approaches, as well as the need to integrate coagulation markers to establish dose-response thresholds of public health relevance.

## 1 Introduction

Cardiovascular diseases (CVDs) remain the most prevalent non-communicable disorders globally, showing an upward trend and accounting for 44% of annual deaths (Munzel et al., 2026). Atherosclerosis, a cornerstone of their development, is characterised by arterial hardening driven by lipid accumulation and a chronic vascular inflammatory process that weakens the fibrous cap (Camacho-Mejorado et al., 2020). As a consequence of vascular injury, rupture of the atherosclerotic plaque exposes thrombogenic surfaces, including tissue factor (TF)-rich subendothelial structures, to circulating blood, thereby initiating the coagulation cascade. This, in turn, triggers the formation of platelet-rich thrombi containing fibrin, leucocytes, and inflammatory mediators (Noonan et al., 2025). Vascular injury also exposes collagen-rich subendothelial matrix components that promote platelet adhesion and contribute to factor XII-mediated contact activation. TF expressed by monocytes, along with TF from the atherosclerotic plaque, initiates the extrinsic coagulation pathway (Asaad et al., 2024). Collagen is also recognised by glycoprotein VI (GPVI), whereas von Willebrand factor (vWF) binds to collagen via the GPIb-V-IX complex, arresting platelets at injury sites. Contact between platelets and the injured vessel wall further activates platelets, releasing paracrine and autocrine signals via adenosine diphosphate (ADP) and thromboxane A2 (TxA2), which promote platelet recruitment and aggregation (Wang et al., 2025). Platelet plug formation is termed primary haemostasis, whilst subsequent fibrin generation constitutes secondary haemostasis. Haemostasis is a physiological process that maintains vascular integrity by balancing clot formation and fibrinolysis, thereby protecting against bleeding (Vilahur and Fuster, 2025). Immunothrombosis, a coordinated action among immune cells, platelets, and coagulation factors, intensifies inflammation, promotes platelet aggregation, and favours a thromboinflammatory state when abnormally activated (Wang et al., 2025). Under this pathological state, atherosclerotic plaque instability is increased, leading to atherothrombosis, the main trigger of myocardial infarction (MI), stroke, and venous thromboembolism (VTE), the leading causes of vascular death (Wendelboe and Weitz, 2024; Abdul Jabbar et al., 2025). Thrombosis, an exaggerated haemostatic response, is evaluated using various haematochemical parameters. Among these, platelet aggregation, platelet count, mean platelet volume (MPV), prothrombin time (PT), activated partial thromboplastin time (aPTT), fibrinogen (FBR), and D-dimer are among the most explored.

Lifestyle-related conditions, including hypertension and metabolic diseases (e.g., diabetes, dyslipidaemia, obesity, and overweight), are well-established risk factors and inducers of endothelial dysfunction, which favour the development of atherosclerosis (Palacios-Valladares et al., 2024; Mlynarska et al., 2025). Family history and behavioural risk factors such as tobacco and alcohol consumption are also among the major drivers of CVDs’ development. Plastics have also been linked to endothelial dysfunction, hypertension, inflammation, lipid dysregulation, and insulin resistance, all of which are recognised CVD risk factors (Palacios-Valladares et al., 2024; Harray et al., 2026). Nevertheless, their effects on thrombosis and haemostasis remain poorly understood.

Acrylamide (ACR), bisphenols, and phthalates are among the most widely used plastic-associated chemicals (PACs), conferring the distinctive properties that make plastics versatile across industries. Hence, PACs are widely used in agriculture, cosmetics and perfumery, construction, food (additives, colourants, and packaging), medical devices, pharmaceutical formulations, toys and childcare, and transport, among many others (Munzel et al., 2025). From these PACs, complex matrices of polymers are formed and combined with stabilisers against ultraviolet (UV) light and heat, as well as chemical additives, flame retardants, biocides, and colourants. UV radiation, chemical oxidation, biodegradation, and physical abrasion can lead to the formation of plastic particles of various sizes from larger plastics (Liu et al., 2020). Microplastics (MPs) are particles with a diameter between 0.10 μm and 5.00 mm, whilst nanoplastics (NPs) are particles with a diameter of less than 0.10 μm. Dietary intake and drinking water are the main exposure pathways for PACs, MPs, and NPs, along with inhalation (air and dust) and transdermal absorption through personal-care products, thermal receipts, and clothing. The full impact of these hazardous cocktails on human health remains unclear.

Thrombosis is a key but often overlooked process in the development of CVDs. Continuous and uncontrolled exposure to platelet aggregation enhancers and prothrombotic compounds represents an under-addressed gap in current CVD prevention strategies. In this review, we examine the influence of plastics on thrombosis using findings from human and preclinical rodent studies, highlighting important gaps in the study of these procoagulant promoters. Changes in haematochemical parameter levels were expressed as percentages relative to the control group, to standardise the presentation of results.

### 1.1 Haemostatic disruption induced by acrylamide, bisphenols, and phthalates

ACR is a hazardous compound and a biomarker of exposure to tobacco and vaping (Hammond et al., 2025). ACR is also a neo-formed contaminant generated during food processing at temperatures above 120 °C and under low-moisture conditions via the Maillard reaction (Toros et al., 2026). This non-enzymatic reaction occurs between amino acids, such as asparagine, and the carbonyl group of reducing sugars, producing desirable flavours, aromas, and colour, as well as ACR. Carbohydrate-rich fried foods (e.g., potatoes), whole cereals and their derivatives (e.g., crisp and toasted bread and biscuits), coffee and cocoa bean roasting, and baked and roasted foods are the main sources of ACR (Yan et al., 2023; Peivasteh-Roudsari et al., 2024). The high electrophilic reactivity of ACR and glycidamide, an intermediate metabolite formed by cytochrome P450 2E1, favours their binding to proteins and their adverse health effects. Accordingly, ACR has been classified as a potent carcinogen and a genotoxic compound by several regulatory agencies, with an estimated average daily intake of 0.60 μg/kg to 4.00 μg/kg body weight (bw) (Yan et al., 2023). Nonetheless, its haematotoxic effects are poorly documented, despite its covalent affinity for haemoglobin, a biomarker of long-term ACR exposure (Merida et al., 2024). Indeed, ACR is among the least studied compounds in haemostasis, underscoring the need for further research in this field.

**FIGURE 1:**
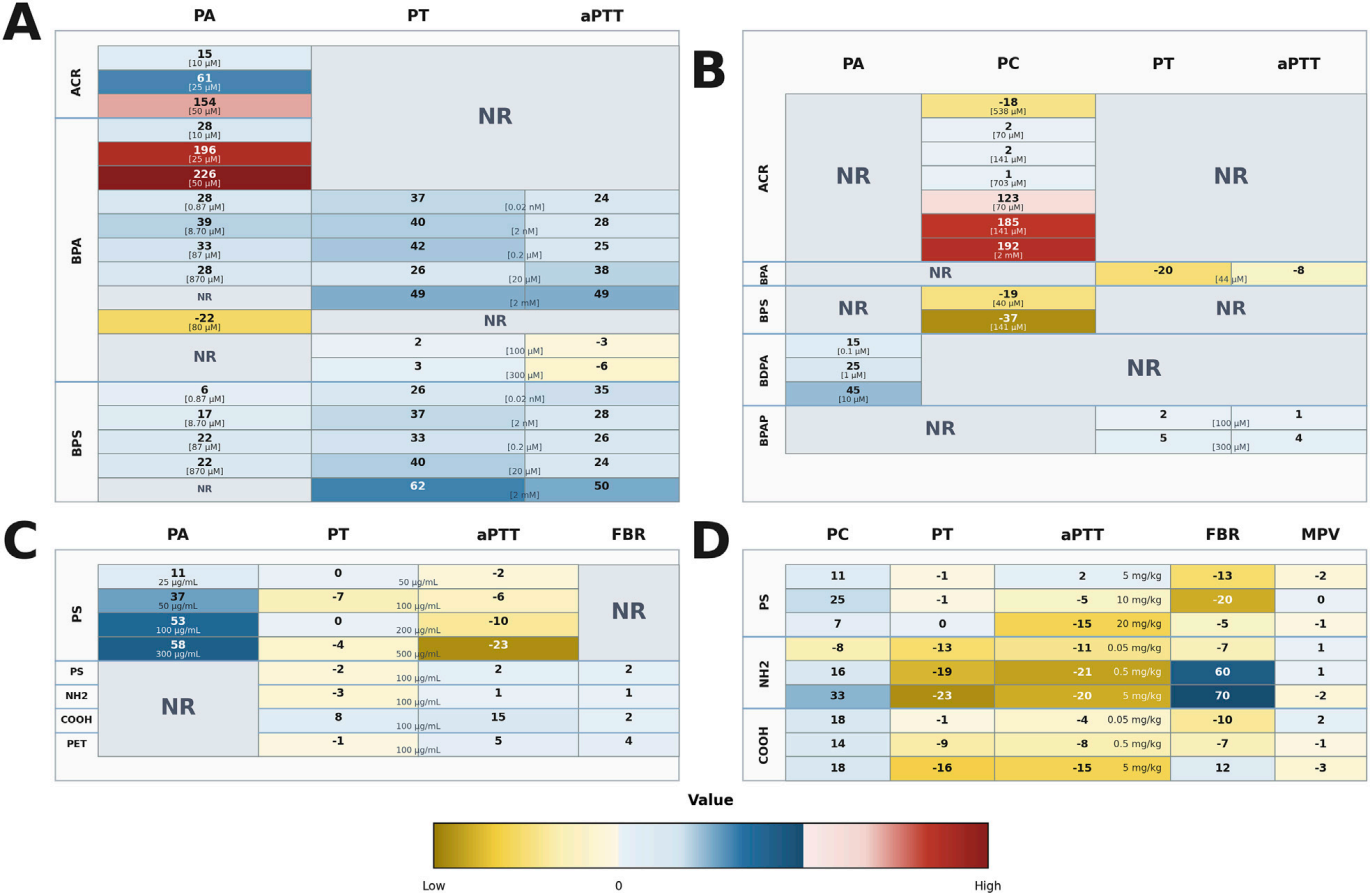
Effects of plastic concentrations on haemostasis relative to controls across several haematochemical parameters. **(A)** Heatmap summarising the effect (%) of acrylamide and bisphenols in human *ex vivo* studies. **(B)** Heatmap summarising the effect (%) of acrylamide and bisphenols based on *in vivo* studies in rats. **(C)** Heatmap summarising the effect (%) of nanoplastics in human *ex vivo* studies. **(D)** Heatmap summarising the effect (%) of nanoplastics based on *in vivo* studies in mice. ACR, acrylamide; aPTT, activated partial thromboplastin time; BPA, bisphenol A; BPS, bisphenol S; BDP, bisphenol A bis (diphenyl phosphate); BPAP, bisphenol AP; COOH, carboxylated nanoplastics; FBR, fibrinogen; MPV, mean platelet volume; NH2, aminated nanoplastics; PA, platelet aggregation; PC, platelet count; PET, polyethylene terephthalate; PS, polystyrene nanoplastics, PT, prothrombin time; NR, non-reported.

Regarding platelet aggregation, to date, only one study has assessed the effect of ACR on human platelets *ex vivo,* reporting a dose-dependent increase in effect (Figure 1A), with significant differences at 50 µM (Burgos et al., 2025). These findings were reinforced by the ACR’s effects on platelet count and MPV in occupationally versus non-occupationally exposed individuals, with a significant increase in occupationally exposed individuals (La Torre et al., 2024). The increase in platelet count has been considered an indicator of thromboinflammation; interleukin-6, a proinflammatory cytokine, has been associated with platelet formation via increased thrombopoietin levels (van der Meijden and Heemskerk, 2019). As a compensatory mechanism for accelerated platelet destruction, these platelets are larger than normal and likely not fully mature (Schmoeller et al., 2017). The larger MPV also indicates that platelets possess enhanced haemostatic properties, both metabolically and enzymatically, that enable them to respond to injury. Previous studies have shown larger MPV in patients with coronary artery disease compared with patients without this condition; MPV is therefore a potential risk biomarker for CVDs (Amsalem et al., 2023). However, larger MPVs have also been documented in comorbidities with CVDs (e.g., diabetes) and in neoplasia, conditions in which ACR is recognised as an effective carcinogen.

The effects of ACR on platelet count from rat studies have been controversial (Figure 1B). In Wistar rats, platelet count decreased significantly (Selimoglu et al., 2025). By contrast, a marked dose-dependent increase in platelet counts (>150%) was observed in the same rat strain compared with the control group (Belhadj Benziane et al., 2019). In F344 rats, a nuanced, non-significant dose-dependent increase in platelet counts was reported in exposed animals compared with controls, accompanied by a larger MPV only at the lowest dose tested (data not shown) (Raju et al., 2015). Doses were similar across these latter two studies (Raju et al., 2015; Belhadj Benziane et al., 2019). Under these conditions, ACR has exhibited species-dependent effects, which have also been associated with the vehicle used and oral bioavailability (drinking > food; ∼42:1), explaining the reported discrepancies (Raju et al., 2015; Belhadj Benziane et al., 2019; Toros et al., 2026).

Bisphenols are key raw materials for the production of polycarbonate plastics, epoxy resins, and polysulfones. Food containers, cooking utensils, drink bottles, packaging materials (e.g., cling film), and the coatings of metal cans, amongst others, contain bisphenols (Harray et al., 2026). These PACs are not covalently bonded to the polymer and are unstable at high temperatures, under UV radiation, or in acidic or alkaline media. Bisphenol A (BPA), the most widely used bisphenol, has been detected in human and animal matrices, with its tolerable daily intake (TDI) set at 0.20 ng/kg/bw (Palacios-Valladares et al., 2024). Given their pathobiological impact, structural analogues, including bisphenol F (BPF), bisphenol S (BPS), and bisphenol AF (BPAF), among others, are increasingly being used. Presently, more than 140,000 new-generation chemicals, including plastic additives, have been synthesised and tested (Eales et al., 2022). The effects of bisphenols on haemostasis have been documented in fewer than ten studies to date, approximately half of which were conducted in humans (Chagas et al., 2021; Abdulkareem, 2022; Akhter et al., 2023; Li et al., 2023; Burgos et al., 2025; Chen et al., 2025; Hrubsa et al., 2025; Mladenka et al., 2025; Hrubsa et al., 2026). These *ex vivo* studies have demonstrated that BPA and BPS increase platelet aggregation (Figure 1A), even at low concentrations, with one controversial study reporting a significant decrease in BPA at 80 μM (Chagas et al., 2021; Burgos et al., 2025; Hrubsa et al., 2025; Hrubsa et al., 2026). These differences have been associated with the agonists used to induce platelet aggregation (i.e., ADP *versus* arachidonic acid), which also affects aggregation stability (Vishalakshi et al., 2021; Kwame et al., 2026). Analogously to BPA and BPS, new alternatives showed prolonged PT and aPTT times, revealing an anticoagulant effect. Notably, 4,4′-thiodiphenol (the parent bisphenol compound) and BPS are formed as a result of the metabolism of the pesticide Temephos, a finding that warrants consideration in tropical regions (Reyes-Chaparro et al., 2020).

Findings from *in vivo* models showed conflicting trends in platelet count (Figure 1B), with a slight increase (∼7%) in Albino rats exposed to BPA, and a clear decrease (between 20% and 36%) in Sprague-Dawley rats exposed to BPS (Abdulkareem, 2022; Li et al., 2023). Regarding platelet aggregation, bisphenol A bis (diphenyl phosphate) showed a concentration-dependent increase, accompanied by platelet adhesion, contractility, and degranulation (Chen et al., 2025). This platelet adhesion has been associated with increased P-selectin, an adhesion molecule with a key role in thromboinflammation, leading to the subsequent activation of specialised platelet receptors (Vishalakshi et al., 2021; Chen et al., 2025). Similar results have been reported with BPAF, in which additional cytoskeletal rearrangements that promote platelet aggregation were observed (Vishalakshi et al., 2021; Burgos et al., 2025). Regarding PT and aPTT, BPA showed shorter times in Wistar rats along with elevated FBR levels and prolonged blood clotting times, reinforcing its procoagulant effects (Akhter et al., 2023). By contrast, bisphenol AP prolonged PT and aPTT times in Wistar Han rats at subacute doses, indicating an anticoagulant effect (Mladenka et al., 2025). Beyond the different strains employed, the tested compound and its doses may also produce divergent effects. The specific effects of each compound illustrate the importance of evaluating bisphenol and even PAC mixtures on haemostasis. In this setting, a previous study demonstrated that leaving bottled water in a car could affect platelet function. These results were associated with higher BPA levels (1.5-fold those in bottles kept at room temperature) and an increase in platelet count (Abdulkareem, 2022).

Phthalates are the least studied compounds regarding haemostasis. These PACs are used in the manufacture of polyvinyl chloride (PVC), which is widely used in pipes, electrical cables, construction, and vehicle industry as well as in food packaging. Similarly to bisphenols, high-molecular-weight phthalates migrate into food and, due to their lipophilic properties, are found in fatty foods, with levels increasing with temperature fluctuations and prolonged use (Palandri et al., 2026). Drinking water also contains phthalates, as these are commonly used in the manufacture of drinking-water containers. Furthermore, cosmetics, lipstick, deodorants, lotions, perfumes, and nail polish contain low-molecular-weight phthalates such as di-butyl phthalate (DBP) and diethyl phthalate (DEP) (Sree et al., 2023). Phthalate esters are not covalently bound and can easily leach into indoor environments, where humans are exposed through inhalation and ingestion of dust particles (Weiss et al., 2018). Toxicological studies indicate that an overall TDI for all phthalates may be inappropriate, suggesting that this parameter should be determined for each phthalate individually (Chang et al., 2017). For mono(2-ethylhexyl)phthalate (MEHP), the active metabolite of di-(2-ethylhexyl) phthalate (DEHP), the daily intake from dust ingestion in adults has been estimated at 28 ng/kg/bw (Weiss et al., 2018). Around 69% of DBP is excreted in urine as mono-*n*-butyl phthalate (MBP), with its parent compound’s daily intake estimated at 0.32 μg/kg/bw in children and 2.3–3.3 μg/kg/bw in adults (Seckin et al., 2009; Dirtu et al., 2013; Li et al., 2019).

Their use as enteric coatings in pharmaceutical formulations and as excipients in controlled-release capsules represents a continuous source of exposure (Sree et al., 2023). Moreover, the indiscriminate use of MEHP and MBP in medical devices such as blood bags, catheters, and dialysis lines has highlighted repeated exposure among hospitalised patients (Biondi-Zoccai et al., 2025). DEHP is the most widely used plasticiser in PVC blood bag systems, migrating into blood and hyperactivating platelets; a negative relationship between its metabolites and cardiometabolic markers has been reported (Harray et al., 2026). Biomonitoring studies have revealed elevated levels of MEHP and MBP in urine and blood samples from patients undergoing intensive care and cardiac surgery. Similar findings have been reported in patients undergoing transcatheter aortic valve replacement and extracorporeal membrane oxygenation (Biondi-Zoccai et al., 2025). Phthalate metabolite concentrations have been positively associated with haematochemical parameters such as platelet count and MPV, whereas PT showed a negative correlation in women (Liu et al., 2025). These trends were also observed in occupationally exposed workers, in whom phthalate metabolites, such as MMP, showed a negative correlation with PT (Tangestani et al., 2026). Other metabolites, such as MEHP and monobenzyl phthalate, were associated with prolonged clotting times. Despite the secondary effects of their exposure, even within medical care settings, these PACs have received limited scientific attention, pointing to a potential research area with significant public health implications.

Findings from *in vivo* studies are particularly scarce; however, chronic dietary exposure to phthalate esters in Wistar Han rats showed a nuanced, dose-dependent increase in PT and aPTT (Lee et al., 2023).

### 1.2 Tiny particles, big consequences: microplastics and nanoplastics

These ubiquitous environmental risk factors arise from complex mixtures of synthetic materials alongside chemical additives; plastic production involves more than 16,000 chemicals (Harray et al., 2026). As a consequence of environmental abrasion, heterogeneous fragmentation patterns may exhibit different physicochemical properties that increase their polarity, hydrophilicity, reactivity, and surface charge, thereby increasing their toxic effects. Among the wide variety of functional groups “expressed” on their surfaces, amine (—NH_2_, carrying a positive charge) and carboxyl (—COOH, negatively charged) have been the most studied. Given that NPs pose analytical challenges for quantification, their study in human biological samples and food is particularly limited; the majority of reports have detected NPs alongside MPs (Wang et al., 2024; Khan and Zaidi, 2025). Consequently, MPs have been the most studied, with publications increasing exponentially over the last 20 years: from one paper in 2006 to more than 5,000 in 2025.

The ubiquitous distribution of both MPs and NPs favours their entry into the human body via several exposure routes, with ingestion being the most important. Beverages, including drinking water, are the main sources of exposure; tea (60 MPs/L) and coffee (43 MPs/L) had the highest MP concentrations, whilst MP concentrations in drinking water ranged up to 1,400 MPs/L (Al-Mansoori et al., 2025; Heo et al., 2025). These plastics have also been found in staple consumer foods such as table salt, sugar, honey, and meat (Espiritu et al., 2024). MPs and NPs have been found in chicken, duck, beef, pork, fish, and seafood; duck showed higher concentrations than chicken, and beef more than pork (Duda and Petka, 2025; Abad-Lopez and Grande-Tovar, 2026). Polypropylene (PP), polystyrene (PS), polyethylene (PE), polyethylene terephthalate (PET), and polyvinyl chloride (PVC) were the most common plastics reported in food and beverages (Al-Mansoori et al., 2025). However, their presence has been associated with geographic region, education level, and lifestyle, including dietary patterns, with the lacto-ovo-vegetarian diet exhibiting the highest MP levels, approaching 70 million particles per day (Duda and Petka, 2025). A cross-sectional study of 36 healthy individuals found that using plastic food containers increased MPs in blood by 20%; PP and PS were the most common microplastics (Lee et al., 2024). An estimated daily average exposure in the United Kingdom has been calculated at 1.65 MPs/kg/bw (Al-Mansoori et al., 2025). In humans, MPs and NPs have been detected in the bloodstream, breast milk, colon, faeces, intestine, kidney, liver, lungs, and placenta (Christodoulou et al., 2026). In the cardiovascular system, MPs and NPs have been detected in blood, heart tissue, and thrombi, and have been associated with adverse cardiovascular effects, attracting more attention than PACs (O' Dowling, 2025). Nonetheless, their potential effects on haemostasis remain poorly characterised and relatively unexplored, highlighting the need for further research in this field. The limited evidence on the effects of MPs on haemostatic parameters showed that the number of MPs increased proportionally with platelet count, PT, aPTT, and FBR levels (Lee et al., 2024; Dmitrowicz et al., 2026; Munzel et al., 2026). In patients with clinical manifestations of atherothrombosis, MPs’ effects on haemostatic parameters promote a prothrombotic state (Wang et al., 2024). The severity of the disease (β∼8, 95% CI: 2.01–13.43) correlated with the number of MPs found in thrombi; the highest concentrations (141.80 μg/g) were detected in MI patients (Wang et al., 2024). These results have been supported by elevated D-dimer levels, a biomarker of active fibrinolysis, indicating a hypercoagulable state. Similar findings, in D-dimer and prolonged thrombin times, were reported in patients with extracranial artery stenosis, a risk factor for stroke (Yu et al., 2024). Other studies have revealed that MPs increase reactive oxygen species (ROS) production, which has been associated with hypertension and cardiac hypertrophy (Du et al., 2025). In particular, PS—NH_2_ accumulates readily in the heart and increases systolic and diastolic blood pressure (Du et al., 2025). Yet, because these studies cannot control for the number of MPs, the findings were stratified into low- and high-MP groups; each study determined its own thresholds, making cross-study comparisons difficult.

Restricted evidence from controlled MP exposure in mice has revealed an inverse-proportional relationship between MP concentration and platelet count (Abdel-Zaher et al., 2023). Interestingly, MPs have shown a stronger binding capacity for fibrin than for platelets, thereby compromising thrombus stability and the subsequent development of vascular diseases (Chen et al., 2022). Rheological effects (morphological alterations, viscosity, and elasticity) on erythrocytes and platelets due to MPs exposure have also been described (Abdel-Zaher et al., 2023). Erythrocyte morphological alterations, including boat-shaped, folded, helmet-shaped, sickle-shaped, and teardrop-shaped cells, affect blood circulation, flow resistance (viscosity), tissue perfusion, and oxygenation. Haematocrit, which is also affected by MP, was closely linked to blood viscosity.

Regarding the effect of NPs on humans, an *ex vivo* study reported that NPs exacerbated the ADP-induced platelet aggregation (Figure 1C) in a dose-dependent manner (Sheng et al., 2024). A similar trend was clearly observed in aPTT, suggesting that NPs could alter the intrinsic coagulation pathway, attenuating thrombin production (Arribas Arranz et al., 2024; Sheng et al., 2024). This effect has been observed in NPs carrying—NH_2_, which have been shown to bind key coagulation factors such as FVII and FIX, thereby decreasing thrombin formation (Oslakovic et al., 2012). By contrast, NPs carrying—COOH activate the intrinsic coagulation pathway via FXII activation. FBR levels were modified differentially depending on the functional group carried by NPs; PET and NPs—COOH showed a major increase, whereas NPs—NH_2_ showed a significant decrease (Arribas Arranz et al., 2024). The decrease in FBR levels by NPs—NH_2_ suggests an increased consumption of this protein and, in turn, an active coagulation state. A previous study demonstrated that NPs—NH_2_ can modify the structure, function, and biological effects of FBR in a dose-dependent manner, promoting blood coagulation (Wang et al., 2023b). PS—COOH has been associated with hypertension, as well as with increased fibrin deposition and favouring platelet aggregation (Christodoulides et al., 2023).

In Sprague-Dawley rats, NP exposure has been shown to increase thrombus weight in a dose-dependent manner (Sheng et al., 2024). Nevertheless, the main findings from *in vivo* models have come from studies in mice. In this setting, NPs showed an increase in platelet count in the BALB/C strain, which was highest with NPs—NH_2_ (Wang et al., 2023a). The effect of NPs on the platelet aggregation exacerbation was consistent with the human *ex vivo* findings. Given their small diameter, NPs have been shown to cross the blood-brain barrier, inducing stroke in rodents, reinforcing their role in CVDs (Huang et al., 2025). Notably, NPs-NH_2_ showed a dose-dependent response in this setting, as well as in PT, aPTT, FBR, and MPV; all these parameters exhibited a significant decrease compared with the control group (Figure 1D). Analogous patterns were observed in NPs—COOH, although less pronounced. These results originate from a single study and should be interpreted with this limitation in mind.

### 1.3 Possible mechanisms

**FIGURE 2:**
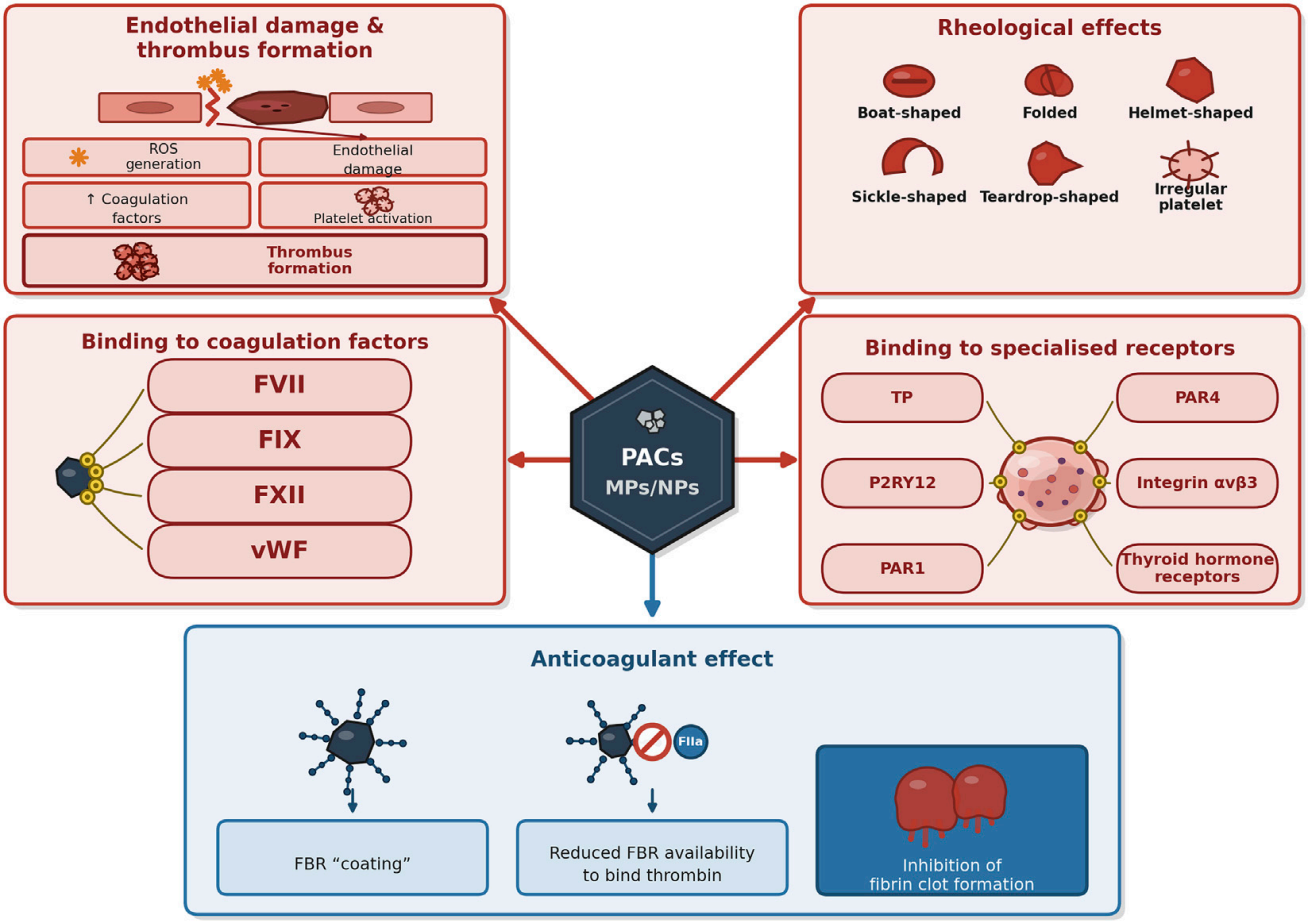
Prothrombotic and anticoagulant mechanisms of plastics. FIIa, thrombin; FVII, coagulation factor VII; FIX, coagulation factor IX; FXII, coagulation factor XII; FBR, fibrinogen; MPs, microplastics; NPs, nanoplastics; PACs, plastic-associated chemicals; PAR1, protease-activated receptor 1; PAR4, protease-activated receptor 4; P2RY12, P2Y-subtype-12 receptor; ROS, reactive oxygen species; TP, thromboxane A2 receptor; vWF, von Willebrand factor.

Both PACs and MPs/NPs showed potential haemostatic effects, revealing non-monotonic dose-response profiles that suggest several underlying, interacting mechanisms shared amongst these endocrine disruptors (Vandenberg, 2014). Overall, all these plastics exerted a dual effect on haemostasis: pro-thrombotic, increasing platelet count and fibrinogen levels, and anticoagulant, prolonging PT and aPTT times (Figure 2). This behaviour suggests that plastics could interfere with both primary and secondary haemostasis (Chagas et al., 2021; Vishalakshi et al., 2021; Fonseca et al., 2022).

From the prothrombotic perspective, the rheological effects of plastics have been associated with plasma lipid levels (Abdel-Zaher et al., 2023; Li et al., 2023). In addition, oxidative stress, leading to increased ROS production and damage to the vascular endothelium, has also been observed, with a consequent increase in procoagulant factors and promotion of platelet activation and clot formation (Palacios-Valladares et al., 2024; Burgos et al., 2025). Plastics bind to targets associated with platelet activation and coagulation, such as Factor VII, FIX, FXII, and vWF, as proposed by several studies (Oslakovic et al., 2012; Chagas et al., 2021; Lett et al., 2021; Abdel-Zaher et al., 2023; Chen et al., 2025). These properties are particularly concerning, as plastics affect haemostasis in multiple ways, including exacerbating platelet aggregation and inducing anticoagulation states (Figure 2).

On the other hand, plastics could directly interfere with platelet function by interacting with specialised receptors. *In silico* models have shown that phthalates, including DEHP and DBP, can interact with a key mediator of platelet activation: integrin αvβ3 (Li et al., 2022). DEHP has also shown potential endocrine-disrupting activity at the thyroid receptor alpha; thyroid hormone dysregulation is associated with hyper- and hypocoagulation states and is closely linked to CVDs (Ellervik et al., 2021; Zughaibi et al., 2022). A recent study highlighted the ability of bisphenols to enhance platelet aggregation through their binding affinities for the TxA2 receptor, the P2Y12 receptor and protease-activated receptors 1 and 4 (Kwame et al., 2026).

The anticoagulant effect has been explained by the coating of plastics by FBR (Figure 2), which interferes with FBR’s binding to thrombin and, in turn, inhibits fibrin generation and clot formation (Tran et al., 2022).

## 2 Conclusion

This mini review provides the first consolidated synthesis of the haemostatic effects of plastics. The collated evidence indicates that all plastics are potential disruptors of both primary and secondary haemostasis. These disturbances are not unidirectional: the same compound could produce prothrombotic effects under some conditions and anticoagulant effects under others. This duality, a feature of endocrine-disrupting chemicals, results from their capacity to interact with multiple haemostatic targets (e.g., coagulation factors, platelet receptors, and fibrinogen) and other mechanisms underlying endothelial dysfunction. The public health implications are substantial. Current CVD prevention strategies do not account for the haemostatic effects of plastics, despite growing evidence that these contaminants have a clear impact on vascular health. Although health policies have regulated the use and consumption of specific PACs to reduce human exposure through food, most exposure still occurs unintentionally. Currently, hazardous combinations of MPs and NPs, along with their intricate mixtures, remain unregulated, owing to perceived insufficient evidence of their detrimental effects on human health. Bisphenols and MPs are the most studied; however, the research gaps to be prioritised in future toxicological approaches involve all plastics and their mixtures, which must be addressed through a One Health approach. Although the evidence remains limited in scope, the findings are consistent, highlighting the need for regulatory attention, particularly for vulnerable populations (i.e., children, adolescents, and young adults). These populations have an accelerated onset of CVDs, rendering them more susceptible.
